# Strong T-cell activation in response to COVID-19 vaccination in multiple sclerosis patients receiving B-cell depleting therapies

**DOI:** 10.3389/fimmu.2022.926318

**Published:** 2022-08-05

**Authors:** Roberto Alfonso-Dunn, Jerry Lin, Vanessa Kirschner, Joyce Lei, Grant Feuer, Michaela Malin, Jiayuan Liu, Morgan Roche, Saud A. Sadiq

**Affiliations:** Tisch Multiple Sclerosis Research Center of New York, New York, NY, United States

**Keywords:** multiple sclerosis, anti-CD20, COVID-19, vaccination, adaptive immunity, B-cell, antibodies, T-cell response

## Abstract

Immunocompromised individuals, including multiple sclerosis (MS) patients on certain immunotherapy treatments, are considered susceptible to complications from severe acute respiratory syndrome coronavirus 2 (SARS-CoV-2) infection and specific vaccination regimens have been recommended for suitable protection. MS patients receiving anti-CD20 therapy (aCD20-MS) are considered especially vulnerable due to acquired B-cell depletion and impaired antibody production in response to virus infection and COVID-19 vaccination. Here, the humoral and cellular responses are analyzed in a group of aCD20-MS patients (n=43) compared to a healthy control cohort (n=34) during the first 6 months after a 2-dose cycle mRNA-based COVID-19 vaccination. Both IgG antibodies recognizing receptor binding domain (RBD) from CoV-2 spike protein and their blocking activity against RBD-hACE2 binding were significantly reduced in aCD20-MS patients, with a seroconversion rate of only 23.8%. Interestingly, even under conditions of severe B-cell depletion and failed seroconversion, a significantly higher polyfunctional IFNγ^+^ and IL-2^+^ T-cell response and strong T-cell proliferation capacity were detected compared to controls. Moreover, no difference in T-cell response was observed between forms of disease (relapsing remitting- vs progressive-MS), anti-CD20 therapy (Rituximab vs Ocrelizumab) and type of mRNA-based vaccine received (mRNA-1273 vs BNT162b2). These results suggest the generation of a partial adaptive immune response to COVID-19 vaccination in B-cell depleted MS individuals driven by a functionally competent T-cell arm. Investigation into the role of the cellular immune response is important to identifying the level of protection against SARS-CoV-2 in aCD20-MS patients and could have potential implications for future vaccine design and application.

## 1 Introduction

Currently approved vaccines designed against the ancestral SARS-CoV-2 (CoV-2) spike protein are efficient in blocking infection, hospitalization and death in healthy individuals (1–3). Adaptive immune responses generated by mRNA-based COVID-19 vaccines (mRNA-1273, BNT162b2) induce the production of high levels of neutralizing antibodies and a strong and sustained T-cell response (4–6). Waning immunity and the rise of new CoV-2 variants of concern have created additional challenges and the need for boosters and potential new vaccine designs. Concern about the level of protection of naïve or vaccinated individuals with congenital or acquired immunosuppression has been raised during the pandemic. A recent CDC study has shown that immunocompromised populations receiving mRNA-based COVID-19 vaccines had lower protection against hospitalization than the general immunocompetent population (77% compared to 90%), although extensive differences were observed depending on type of disease and treatments (7). In addition to higher susceptibility to complications, protracted infections in immunodeficient individuals can potentially lead to the accumulation of CoV-2 mutations and the rise of new variants of concern (8–10). Overall, these and other factors highlight the need for a better understanding of the immune responses to CoV-2 in immunocompromised individuals.

Patients with multiple sclerosis under B-cell depleting anti-CD20 treatment (aCD20-MS) and other disease-modifying therapies (DMTs) are susceptible to severe acquired immunosuppression and prone to infections (11, 12). Moreover, infections might be responsible for disease exacerbation and only non-live vaccines are currently recommended for the application of immunizations (13). Under anti-CD20 treatment, the induced depletion of immature and mature/naïve B-cells can attenuate the production of antibodies needed for the neutralization of incoming pathogens and blocking of disease onset (14, 15). An important unknown is whether the remaining adaptive immune response, or other unaffected immune arms, can still provide protection against microbe infections. A better understanding of this limitation is essential for defining the level of protection of aCD20-MS patients in the context of the current COVID-19 pandemic, including immunological protection induced by CoV-2 infection or in response to the application of the various vaccines available.

A number of studies have detected higher susceptibility to severe COVID-19 in unvaccinated MS patients on several therapy treatments, including anti-CD20 (16–18). In contrast, other published reports have found no association between treatment with DMTs and disease severity (19, 20). The difficulty of controlling for cofounding factors might explain, at least in part, the reason for this discrepancy. Upon CoV-2 infection or COVID-19 vaccination, aCD20-MS patients have a reduced capacity to develop antibodies against viral proteins (21–25). Because neutralizing antibodies are directly associated with vaccine efficacy (26, 27), lower antibody protection explains recent reports of higher risk of breakthrough infection in vaccinated aCD20-MS patients (28, 29). For this group of immunocompromised individuals, the level of protection against severe COVID-19 disease provided by current vaccines is still unknown.

T-cell responses are recognized as important cellular controllers against intracellular pathogens, including CoV-2. Although less studied than the role of neutralizing antibodies, an important contribution of cellular responses to clinical protection against CoV-2 infection is supported by several evidences (30, 31). In the context of primary infection, asymptomatic COVID-19 is characterized by the mounting of early, strong and coordinated T-cell and antibody responses (32–34). A durable T-cell response up to 1 year after CoV-2 infection has been observed (35), and detection of T-cell reactivity against similar coronavirus (SARS-CoV-1) has been possible many years after exposure (36). Even in the context of new variants of concern, T-cell-mediated immunity could be of more significance to responding to infection compared to antibodies because of the ability to recognize a broader range of viral epitopes, including those shared among the different variants (37–39). In addition, CD4^+^ T-cells generated in response to seasonal coronavirus infections have been shown capable of cross-reacting against several CoV-2 proteins (40, 41). A role for these pre-existing T-cells in immune protection is supported by studies showing the presence of highly responsive cross-reactive cells in seronegative health care workers with abortive infections (42), and a positive correlation with increased CoV2-specific CD4^+^ T-cell and antibody responses to COVID-19 mRNA vaccines (41, 43). Interestingly, additional proof of an important role for T-cells in protection against severe infection comes from a study examining patients with hematologic cancer, showing the generation of a T-cell response and disease control even in the absence of neutralizing antibodies (44). Although much research on the cellular response to CoV-2 infection has been done, a clear challenge in understanding the specific roles of T-cell responses in the protection against COVID-19 is the inherently intertwined nature of the adaptive immune system. Studying cases in which the cellular and humoral responses are “decoupled” could offer unique opportunities to better understand the specific contribution of each arm (45).

The effects of COVID-19 vaccination on the development of a competent T-cell response in aCD20-MS individuals is unclear, with recent studies mostly focused on relapsing remitting-MS patients treated with Ocrelizumab. Published data indicates higher (21, 46, 47), lower (48), or no change (23, 49) in vaccine-elicited cellular immune responses measured by using different methodologies for the *ex vivo* detection of cytokine^+^-releasing T-cells. Knowing which arms of the immune response are functionally active is essential in determining the level of protection of vaccinated aCD20-MS patients against CoV-2 infection and disease, and in reviewing the design and application of future vaccines.

The present single-center study characterizes the humoral and cellular immunological responses developed in COVID-19 vaccinated aCD20-MS patients. To achieve this, peripheral blood samples were collected from patients with relapsing remitting (RRMS) and progressive (PMS) forms of disease, treated with either Rituximab or Ocrelizumab anti-CD20 monoclonal antibodies and recipients of a two-dose mRNA-based COVID-19 vaccine regimen in the past 6 months. In response to vaccination, lower anti-spike receptor binding domain (RBD) IgG antibody titers and RBD-human cellular receptor angiotensin-converting enzyme 2 (hACE2) blocking activity were detected in aCD20-MS compared to a healthy control cohort. Surprisingly, even with marked B-cell depletion and low antibody titers in peripheral blood, aCD20-MS patients display a significantly higher spike-specific T-cell response compared to healthy controls as measured using *ex vivo* cytokine release and cellular proliferation assays. Importantly, no differences were observed when comparing different forms of diseases (RRMS vs PMS), anti-CD20 treatments (Ocrelizumab vs Rituximab), or mRNA-based vaccine received (mRNA-1273 vs BNT162b2). These results suggest the generation of a partial adaptive immune response to COVID-19 vaccination in B-cell depleted MS patients driven by a functionally competent T-cell arm. Investigation into the role and durability of this cellular immune response should be considered for a more complete characterization of the correlates of protective immunity against CoV-2 in aCD20-MS patients.

## 2 Materials and methods

### 2.1 Human subjects and study design

Collection of blood samples from vaccinated aCD20-MS patients with relapsing remitting- and progressive- forms of multiple sclerosis, as well as from vaccinated healthy controls and CoV-2 unexposed controls was carried out at Tisch Multiple Sclerosis Research Center of New York under institutional review board (IRB)-approved protocol #20211254. Unexposed controls reported no PCR-confirmed CoV-2 infection at the moment of sample collection and were determined to be seronegative using the serological assays described in section 2.3. Individuals receiving a full 2-dose mRNA-based [BNT162b2 (Pfizer-BioNTech) or mRNA-1273 (Moderna)] vaccination regimen with a 3- to 4- week dosing interval were considered in this study. Also included were a reduced number of individuals injected with one dose of the adenoviral vector-based Ad26.COV.2.S vaccine (Johnson & Johnson-Janssen). MS patients received their first vaccination dose an average of 114.5 days [IQR: 81-138.5] after their last anti-CD20 infusion treatment. All samples were collected within 6 months after full immunization. Vaccinated individuals with previous infection or with vaccine boosters, and MS patients receiving their first anti-CD20 infusion after vaccination were not included. For all participants, the first vaccine injection was applied between January and June of 2021 and the period of sample collection lapsed from March to November of the same year. Medical and drug information as well as infection and vaccination history were extracted from medical records and questionnaires. All participants signed written informed consent prior to enrollment. See **Table 1** for clinical characteristics.

**Table 1:** Clinical characteristics of study participants.

	HC (n=34)	aCD20-MS (n=43)	P value^a^
Age, mean years [range]	34.7 [23-59]	53.9 [28-82]	
Relapsing Remitting (RRMS)		48.4 [28-74]	<0.001
Progressive (PMS)		63.2 [35-82]	<0.001
**Gender, *n* (%)**			
Male	8 (23.5)	16 (37.2)	0.198
Female	26 (76.5)	27 (62.8)	
**MS type, *n* (%)**			
RRMS		27 (62.8)	
PMS		16 (37.2)	
**anti-CD20 therapy, *n* (%)**			
Ocrelizumab		27 (62.8)	
Rituximab		16 (37.2)	
**Vaccine type, *n* (%)**			
BNT162b2 (Pfizer-BioNTech)	33 (97.1)	22 (51.2)	
mRNA-1273 (Moderna)	1 (2.9)	18 (41.8)	
Ad26.COV2.S (J&J-Janssen)		3 (7)	
**Last infusion to 1^st^ dose vaccination interval, median days [IQR]**		114.5 [81-138.5]	
RRMS		117.5 [89-139.2]	
PMS		114.5 [71.2-139.2]	
**Full vaccination to collection interval, median days [IQR]**	63 [59.7-90]	64 [42-93]	0.406
**CD3^+^/4^+^/8^+^ absolute count, mean cell/μL [reference range]**			
CD3^+^		1250 [721-2704]	
CD4^+^		922 [423-1614]	
CD8^+^		325 [143-1039]	
**CD4^+^/CD8^+^ ratio [reference range]**		3.9 [0.9-4.3]	
**CD19^+^ absolute count > 20 cell/μL,** *n* (%)		3 (7)	

Clinical characteristics of vaccinated multiple sclerosis patients treated with anti-CD20 (aCD20-MS) and healthy control (HC) cohorts. IQR, interquartile range.

a Statistical significance of differences in the age and full vaccination to collection interval of the groups was assayed by Mann-Whitney U tests; statistical significance of differences in the distribution of female/male was assayed with Chi-square test.

### 2.2 Serum and peripheral blood mononuclear cells isolation

Blood samples from MS patients were obtained immediately before their scheduled anti-CD20 re-administration infusion treatment (with Rituximab or Ocrelizumab). Lymphocyte count for each patient was performed before sample collection. For each individual, peripheral blood was drawn into a Vacutainer CPT™ Sodium Heparin tube (BD Biosciences, #362753) and a Vacutainer SST™ (BD Biosciences, #367988) for PBMCs and serum isolation, respectively. All sample processing was performed within 30 minutes after blood collection. After counting, cells were resuspended with CryoStor CS10 (STEMCELL, #07930) and cryopreserved at 1.5-3x10^6^ cells/mL concentration in liquid nitrogen until use for T-cell response assays. Serum samples were frozen immediately at -80^°^C. For one of the aCD20-MS patients, only PBMCs were obtained and no humoral response analysis was performed.

### 2.3 Determination of anti-spike antibody titers and hACE2 binding blocking activity

#### 2.3.1 SARS-CoV-2 S protein RBD immunoglobulin G ELISA

IgG antibodies against the receptor binding domain (RBD) of CoV-2 spike S1 subunit were detected using an ELISA-based serology assay. One hundred microliters of recombinant CoV-2 RBD protein (Raybiotech, #230-30162-100) at 0.5 µg/mL concentration was added into a 96-well polystyrene – high binding surface EIA/RIA Assay Microplate (Corning, #CLS3361) and incubated O.N. at 4°C. Wells were washed three times and blocked with 250 µL PBS + 1% non-fat dry milk. Serum samples were prepared at 1:1600 dilution in PBS + 1% non-fat milk and 100 µl added into each well considering technical duplicates. Controls added in each plate include blank/secondary only control and a positive serum sample control from a participant that was used for validation and for normalization of all the other test samples on the same plate. Samples were incubated for 1 hour at room temperature and plates washed 5 times with 275 µL PBST. For antibody detection, HRP-conjugated Goat anti-Human IgG (H+L) cross-adsorbed secondary antibody (ThermoFisher, #A18811) was diluted at 1:20000 in PBS + 1% non-fat milk, 100 µl added to each well, and incubated for 1 hour. After 5 washes with PBST, plates were incubated with TMB Substrate Solution (ThermoFisher, #N301) for 10 min prior to stopping the reaction with 0.16 M sulfuric acid Stop Solution (ThermoFisher, #N600). Plates were then read for absorbance at 450 nm (BioTek Synergy HT microplate reader) within 30 min of stopping the reaction. Optical density (OD) is calculated as the absorbance at 450 nm and data is presented as relative to a known positive serum sample control. Limit of sensitivity was set by measuring antibody levels from archival (pre-2020) and more recent CoV-2 unexposed samples (n=51), and a mean + S.D. value was used as the lower limit point.

#### 2.3.2 hACE2 binding blocking assay

Antibodies blocking the binding of SARS-CoV-2 RBD to human ACE2 were detected using the commercial SARS-CoV-2 Surrogate Virus Neutralization Test cPass kit (GenScript Biotech). All samples were tested in duplicates and percent inhibition of RBD-hACE2 binding was calculated using the following equation: $\%\;inhibition=\left(1-\left[\frac{OD\;of\;serum+RBD}{OD\;of\;negative\;control+RBD}\right]\right)$ . As described by the cPass kit, a cut-off of 30% and above was used to determine positive RBD hACE2 inhibition.

### 2.4 FluoroSpot assay for cytokine^+^ T-cell response analysis

A FluoroSpot kit to simultaneously measure the secretion of human IFNγ and IL-2 at the single-cell level was used according to the manufacturer’s protocol (ImmunoSpot). PBMCs were plated on FluoroSpot M96 well plates at 2-3 x10^5^ cells per well and incubated for 24 hours with DMSO negative control (0.4%), a full overlapping 15-mer SARS-CoV-2 spike peptide pool (JPT, #PM-WCPV-S-1; 1 µg/mL) and CEFX positive control peptide pool (JPT, #PM-CEFX-1; 1 µg/mL). For titration experiments using the spike peptide pool, a concentration range between 2.5 to 10^-4^ µg/mL was considered. Co-stimulation with anti-CD28 (ImmunoSpot; 100 ng/mL) was added for all incubations. Spots were counted using a Cellular Technology Limited S6 Universal Analyzer and data processed with ImmunoSpot^®^ 7.0 software. Counting parameters were set optimally for each filter individually and then a pairing algorithm using the center of mass distance for each spot was used to determine co-expressors. Mean spot forming units (SFU) obtained from DMSO incubations were subtracted from the mean of duplicate or triplicate test wells to generate normalized readings expressed as ΔSFU per million PBMCs. Only samples with 20 or more SFU per 10^6^ cells in CEFX stimulations were considered in this study. The value of (mean + 2xS.D.) obtained from unexposed individuals (pre-2020 and more recent unvaccinated/non-infected individuals; n=18) was used as the lower limit to indicate a positive response in the test group (**Figure S1**).

### 2.5 T-cell proliferation assay

Thawed PBMCs were stained with Carboxyfluororescein 6 succinimidyl ester (CFSE) dye (ThermoFisher, #C34554; 0.5 µM). After a 20-minute incubation at 37^°^C, the labeling reaction was stopped by adding 5 volumes of cold CTS OpTmizer T-cell expansion SFM medium (ThermoFisher, #A1048501) containing 5% FBS. Cells were resuspended with FBS-free buffer and 2x10^5^ cells were plated in M96 round-bottom wells for T-cell proliferation assays. Stimulations were carried out in duplicate wells using a full overlapping 15-mer SARS-CoV-2 spike peptide pool (JPT, #PM-WCPV-S-1; 1 µg/mL) and DMSO negative control (0.4%). After 6 days of incubation, cells were collected and the percentage of proliferating CD4^+^ and CD8^+^ cells presenting CFSE-low signal was detected using flow cytometry. Mean values from DMSO incubations were subtracted from the mean of test groups, and presented as % CD^+^ CFSE-low. A positive response was defined as one with % CD^+^ CFSE-low cells at least 1.5x greater than background and greater than 0.2% CD^+^ CFSE-low cells in magnitude following background subtraction.

### 2.6 Flow cytometry

To evaluate cell surface markers on proliferating T-cells, PBMCs were incubated for 30 minutes at 4°C with PE/Cyanine7 anti-CD3 (clone SK7; Biolegend, #344815), PE-CF594 anti-CD4 (clone RPA-T4; BD Biosciences, #562316), and APC anti-CD8 (clone SK1; Biolegend, #980904) prepared in FACS buffer containing 2% FBS at the recommended concentrations. For all experiments, cells were stained with the LIVE/DEAD Fixable Violet Dead cell stain kit (ThermoFisher, #L34963) following the recommended protocol and only viable cells were included in the analysis. Flow cytometry was performed on a FACSAria II Flow Cytometer machine (BD Biosciences). Forward and side scatter gates were used to discriminate doublets and debris. See **Figure S6** for flow cytometry gating strategy. All data analysis was done using FlowJo software (BD Biosciences).

### 2.7 Quantification and statistical analysis

All quantifications and statistical analyses were performed with GraphPad Prism 9 and FACSAria II. Correlation coefficients between humoral (relative anti-RBD IgG OD) and cellular (ΔSFU per million PBMC) responses were quantified by the Spearman rank coefficient. Statistical analysis used the Mann-Whitney U (Wilcoxon) test for comparison between healthy controls and aCD20-MS cohorts and between the different aCD20-MS subgroups. The chi-square test was used to test between group differences for the categorical variables. All tests were two-tailed, and a P-value <0.05 was considered to indicate a statistically significant difference. Box plots representing mean and 95% confidence interval (CI) are shown in the figures. Parameters are stated in the text, figures, and figure legends. Where indicated, asterisks denote statistical significance as follows: *P < 0.05; **P < 0.01; ***P < 0.001.

## 3 Results

### 3.1 Study characteristics

A single-center prospective study was organized for analysis of the development of adaptive immune responses in vaccinated, antiCD20-treated [Ocrelizumab (n=27) or Rituximab (n=16)] multiple sclerosis patients [RRMS (n=27) or PMS (n=16)]. MS individuals received the first [mRNA-1273 (n=22) or BNT162b2 (n=18)] or only [Ad26.COV.2.S (n=3)] vaccination dose an average of 114.5 days [IQR: 81-138.5] after their last anti-CD20 infusion treatment. Samples were collected for antibody and T-cell response analysis at a median of 64 days [IQR: 42-93] post-full vaccination. A vaccinated healthy control cohort (n=34) and unexposed individuals including archival (pre-2020) samples were used as controls. The immunized control group is composed of health care workers of a significantly younger age who almost exclusively received mRNA-based BNT162b2 vaccination (33 of 34). Normal T-cell count values within reference range but with highly reduced CD19^+^ B-cell levels (< 20 cells per µL) were detected for most aCD20-MS patients in routine blood sample analysis performed before treatment infusion and sample collection. **Table 1** indicates details of the clinical characteristics of participants.

### 3.2 Defective humoral response to COVID-19 vaccination in antiCD20-treated MS patients compared to healthy controls

**Figure 1:**
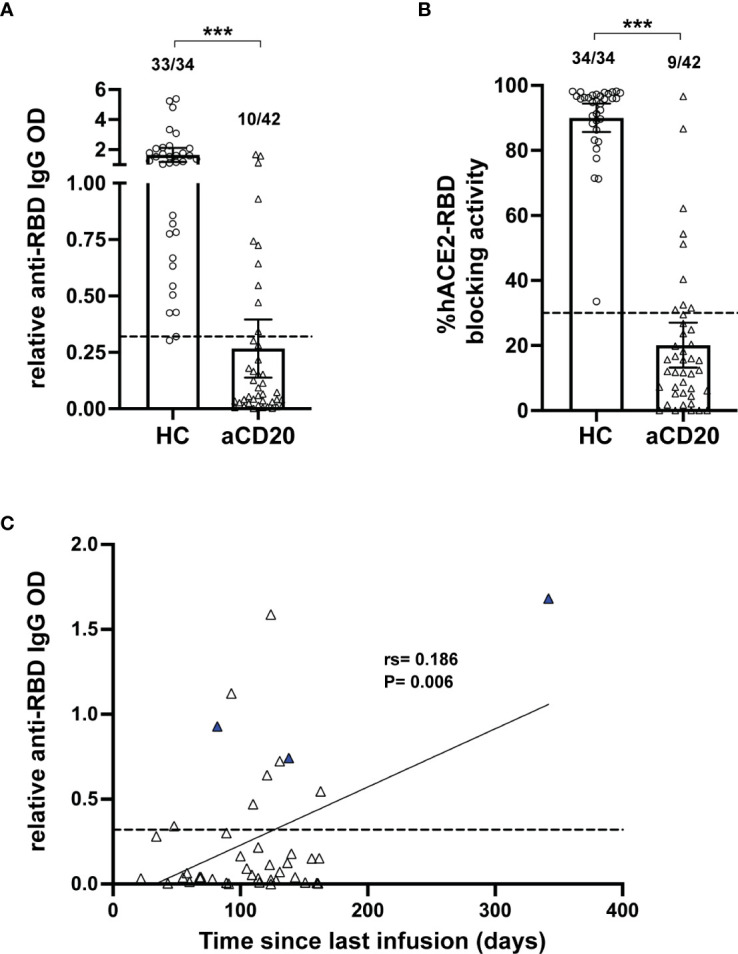
Deficient humoral response in aCD20-MS compared to healthy controls. **(A-C)** Humoral response analysis performed with serum samples collected within 6 months after vaccination of healthy controls (open circles, n=34) and aCD20-MS patients (open triangles, n=42). Dots represent individual data points. **(A)** Comparison of anti-spike RBD IgG antibody titers from serum samples of healthy controls and aCD20-MS patients expressed as relative OD values. Box plots represent mean and 95% CI. Dotted line indicates limit of sensitivity (0.32) and represents mean + S.D. positivity cut-off obtained from unexposed individuals (n=51). Fractions of samples above limit of sensitivity are indicated on top of each dataset. ***P < 0.001; Mann-Whitney U tests. **(B)** Antibody-induced blocking activity of hACE2 binding to RBD was determined from serum samples using the SARS-CoV-2 Surrogate Virus Neutralization Test cPass™ kit (GenScript) and expressed as % hACE2-RBD blocking activity. A percentage of 30% was used to determine positive RBD-hACE2 inhibition. Fractions of samples above positivity threshold are indicated on top of each dataset. ***P < 0.001; Mann-Whitney U tests. **(C)** Linear regression of the ratio of relative anti-RBD IgG OD value and time between last anti-CD20 infusion therapy and first vaccination dose of aCD20-MS patients showing significant correlation between IgG titers and time post-last infusion. Blue triangles represent patients with B-cell counts higher than 20 cells per µL. Dotted line indicates limit of sensitivity.

Anti-CD20 treatment in multiple sclerosis patients is known to decrease antibody titers in response to infections and vaccine applications, including in the context of COVID-19 (21–24). In order to determine antibody levels generated in the study cohorts, we focused on antibody binding to the receptor binding domain (RBD) from subunit 1 of the CoV-2 spike protein. This domain is essential for binding of the virus to the human cellular receptor angiotensin-converting enzyme 2 (hACE2) that triggers host cell infection and represents a major target of neutralizing antibodies (50). A significant decrease in the relative anti-RBD IgG OD (HC, mean 1.65; aCD20-MS, 0.27; P<0.001) and hACE2-RBD percentage blocking activity (HC, mean 90.02; aCD20-MS, 20.27; P< 0.001) was observed in the aCD20-MS cohort compared to healthy controls (**Figures 1A, B**), with only 10/42 (23.8%) of MS patients with antibody titers above the limit of sensitivity and 9/40 (22.5%) showing positive blocking activity (compared to 97.1% and 100% in the healthy control cohort). As shown by others (25, 46), higher antibody levels were detected with longer interval times between beginning of vaccination and last anti-CD20 infusion received (**Figure 1C**). Overall, aCD20-MS individuals have a defective humoral response to COVID-19 vaccination due to severe induced B-cell deficiency. Furthermore, allowing sufficient time between therapy and vaccination can increase the chances of seroconversion.

### 3.3 Increased IFNγ^+^, IL-2^+^ and polyfunctional IFNγ^+^/IL-2^+^ T-cell responses in aCD20-MS compared to healthy controls

Antigen-specific adaptive T-cell immune responses represent an essential arsenal to fight against infections and are known to play important roles during CoV-2 infection (51, 52). Moreover, current COVID-19 vaccines are known to induce a strong poly-specific T-cell response mediated by IFN^+^ or IL-2^+^ CD8^+^ and CD4^+^ Th1-cells (4). Recent published data have shown conflicting results with reported increased, lower or similar T-cell responses in vaccinated aCD20-MS patients compared to healthy controls (21, 23, 46–49).

**Figure 2:**
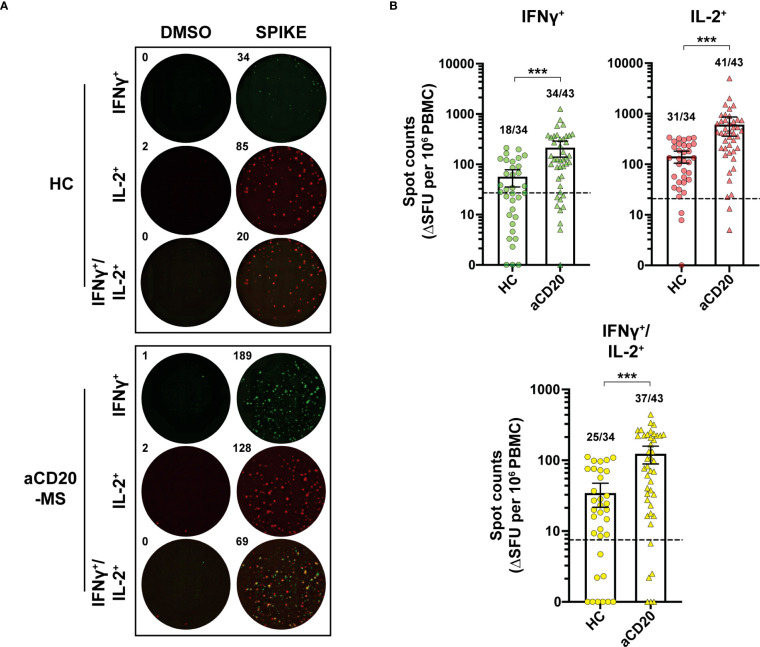
Increased IFNγ^+^, IL-2^+^ and polyfunctional IFNγ^+^/IL-2^+^ T-cell response in aCD20-MS compared to healthy control group. **(A, B)** PBMCs from healthy controls and aCD20-MS patients were placed into IFNγ and IL-2 FluoroSpot plates (ImmunoSpot) and stimulated with DMSO negative control (0.4%) and a 15-mer full overlapping spike peptide pool (1 µg/mL) for 24 hours. **(A)** Representative IFNγ^+^ (green), IL-2^+^ (red) and IFNγ^+^/IL-2^+^ (yellow) FluoroSpot data after antigen incubation of PBMCs from one aCD20-MS patient and one healthy control individual. **(B)** Comparison of the magnitude of IFNγ^+^ (green), IL-2^+^ (red) and IFNγ^+^/IL-2^+^ (yellow) T-cell response in healthy controls (circles, n=34) and aCD20-MS patients (triangles, n=43). Data are DMSO-negative control subtracted and presented as ΔSFU per million PBMC. Dotted line indicates mean + 2xS.D. threshold obtained from unexposed controls (IFNγ^+^= 27, IL-2^+^= 20.8, IFNγ^+^/IL-2^+^= 7.5) (see **Figure S1**). Fractions of samples above threshold are indicated on top of each dataset. Box plots represent mean and 95% CI. ***P < 0.001; Mann-Whitney U tests. See also **Figures S1**–**S4**.

To determine the levels of spike-specific T-cell activity in this study’s groups, *ex vivo* stimulation analysis measuring cytokine^+^ release activity using a FluoroSpot assay was performed. First, the number of T-cells releasing IFNγ^+^, IL-2^+^ and polyfunctional IFNγ^+^/IL-2^+^ was measured simultaneously using a FluoroSpot assay (**Figure 2A**). After exposure of PBMCs to a 15-mer peptide pool overlapping the full CoV-2 spike protein (1 µg/mL), a significant increase in spot counts expressed as ΔSFU per million PBMC was detected in the aCD20-MS group (IFNγ^+^, mean 214.4; IL-2^+^, 608.3; IFNγ^+^/IL-2^+^, 123.5) compared to the healthy control cohort (IFNγ^+^, mean 56.7; IL-2^+^, 143.4; IFNγ^+^/IL-2^+^, 34.5; P<0.001) (**Figure 2B**). Using unexposed individuals to establish a positivity cut-off (**Figure S1**), cytokine release above threshold was observed in 79.1% (IFNγ^+^), 95.3% (IL-2^+^), and 86.0% (IFNγ^+^/IL-2^+^) of aCD20-MS individuals, compared to 52.9%, 91.2%, and 73.5% in the control cohort. Overall, 95.3% of aCD20-MS expressed at least one cytokine compared to 91.2% in controls. Titration with decreasing concentrations of the spike peptide pool (2.5 to 10^-4^ µg/mL) showed higher response from aCD20-MS PBMCs also at lower concentrations (**Figure S2**), suggesting that the stimulated antigen-specific T-cells do not represent low affinity cells.

Importantly, analysis within the aCD20-MS cohort detected no significant difference in cytokine^+^ T-cell response activity when comparing forms of MS disease (RRMS vs PMS), anti-CD20 monoclonal antibody treatments (Ocrelizumab vs Rituximab) and mRNA vaccine received (mRNA-1273 vs BNT162b2) (**Figure S3**). In addition, a significantly higher T-cell response was also observed in the aCD20-MS cohort compared to healthy control group when only considering BNT162b2-vaccinated individuals (**Figure S4**).

### 3.4 Correlative analysis

**Figure 3:**
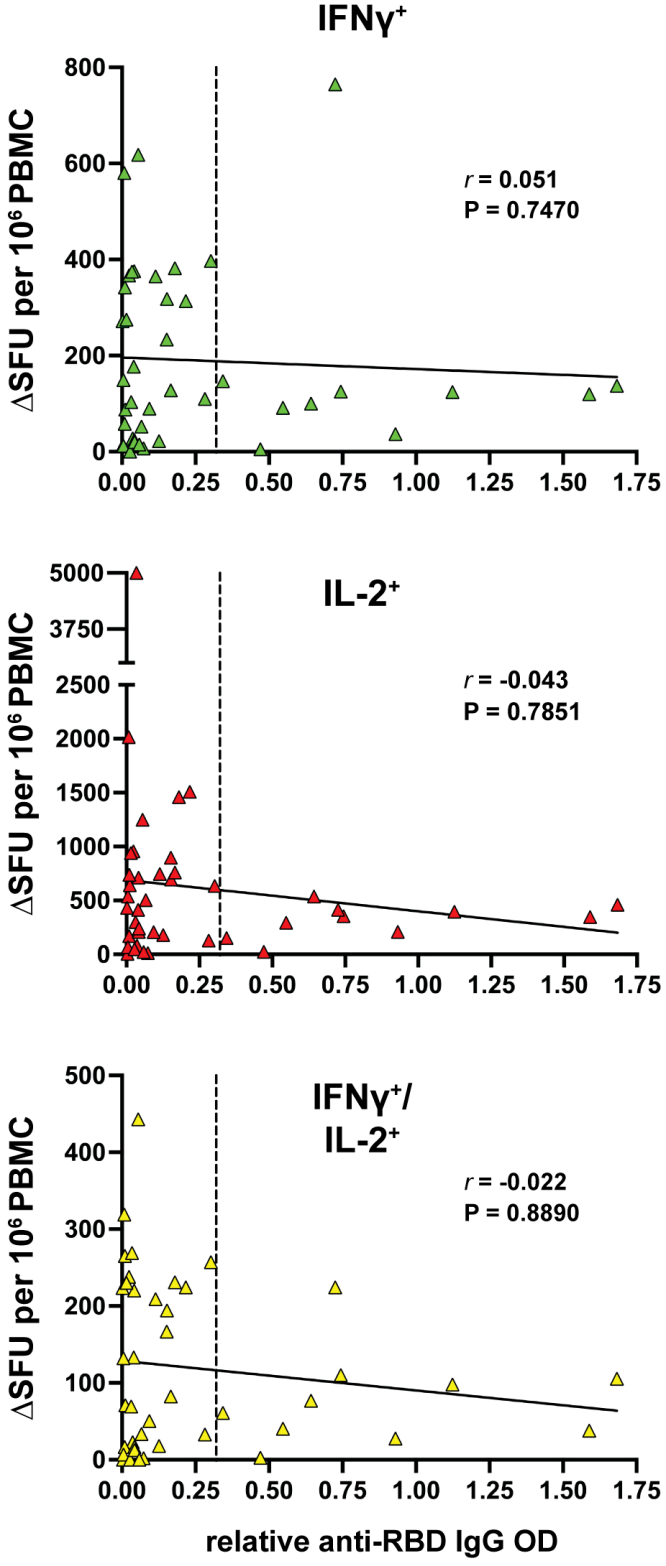
Lack of correlation between cytokine^+^ T-cell and humoral responses in MS patients treated with anti-CD20. Spearman’s correlation between IFNγ^+^ (green), IL-2^+^ (red) and IFNγ^+^/IL-2^+^ (yellow) T-cell response and the relative anti-RBD IgG OD values of aCD20-MS patients (n=42). *r* coefficient and P value are included for each correlation. The bold, continuous line indicates the regression line and the vertical dotted line highlights limit of sensitivity for antibody titers.

Overall, the data presented here highlights a surprisingly strong active T-cell response in aCD20-MS patients even in the presence of a compromised humoral response to COVID-19 vaccination. Interestingly, previous studies have highlighted an inverse association between the adaptive immune responses in vaccinated aCD20-MS patients, with individuals lacking anti-RBD IgG production showing a more robust CD8^+^ T-cell response (21, 46). In the present study, no correlation at the individual level was detected between the number of cytokine^+^ T-cells and relative anti-RBD IgG antibody titers (**Figure 3**), suggesting divergent responses of the humoral and cellular immunity occurring in these individuals.

As mentioned above, aCD20-MS patients in this study are significantly older than the healthy control cohort (**Table 1**). To determine whether an association exists between age and the measured humoral and cellular immune responses in aCD20-MS patients, linear regression analysis was performed. As presented in **Figure S5**, no significant correlation with age of the relative anti-RBD IgG antibody levels and IL-2^+^ T-cell responses was observed.

### 3.5 Higher *ex vivo* T-cell expandability in aCD20-MS compared to healthy controls

**Figure 4:**
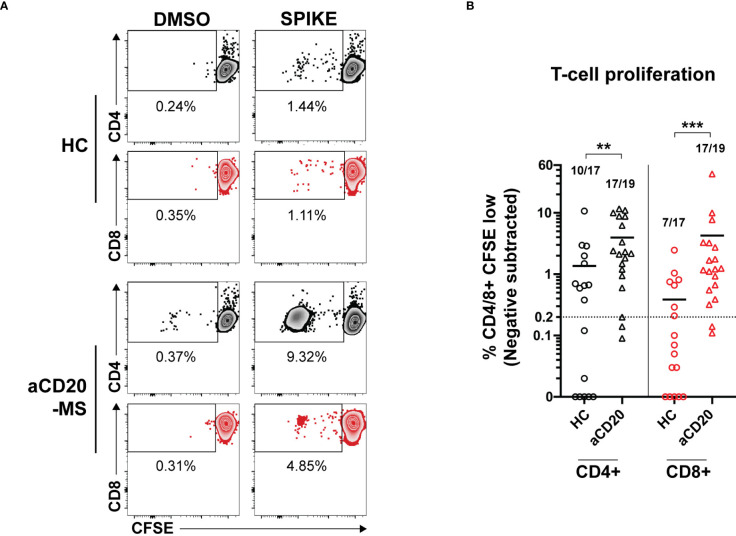
Increased *ex vivo* T-cell proliferation after stimulation with CoV-2 spike peptide pool in aCD20-MS patients compared to healthy control group. **(A, B)** PBMCs from healthy controls (n=17) and aCD20-MS patients (n=19) were stained with CFSE dye and stimulated with DMSO negative control (0.4%), and full overlapping spike peptide pool (1 µg/mL) for 6 days. **(A)** Representative flow cytometry data for the detection of CFSE-low proliferating CD4^+^ (grey) and CD8^+^ (red) T-cells after antigen incubation of PBMCs from one aCD20-MS patient and one healthy control individual. See also **Figure S6**. **(B)** Comparison of magnitude of proliferative CD4^+^ (black) and CD8^+^ (red) T-cells responses after exposure to overlapping spike peptide pool. Data is presented as %CD4/8^+^ CFSE-low, with DMSO negative control values subtracted. Only test samples with %CD4/8^+^ CFSE-low value above 1.5X negative control and higher than 0.2% after negative subtraction were considered positive. Fractions of samples above positivity cut-off are indicated on top of each dataset. Horizontal lines inside graph represent mean values. Calculated P values are as follow: **P < 0.01, ***P < 0.001; Mann-Whitney U tests.

Upon antigen-specific stimulation, T-cells acquire an active cytokine secretion phenotype and undergo a strongly induced cellular proliferative response (53). After detecting higher cytokine^+^ releasing T-cells in aCD20-MS, we wanted to know if induced T-cells are capable of proliferating after stimulation with spike protein even under conditions of B-cell deficiency. To this end, a subgroup of samples representing all cytokine^+^ T-cell response quartiles in both aCD20-MS and control cohorts was selected. PBMCs were stained with CellTrace CFSE dye (0.5 µM) and the percentage of proliferating CFSE-low CD4^+^ and CD8^+^ T-cells was determined after 6 days of peptide stimulation using flow cytometry (**Figures 4A** and **S6**). A higher CD4^+^ and CD8^+^ T-cell proliferation rate was observed in PBMCs from aCD20-MS patients (%CD4+ CFSE-low, mean 3.9; %CD8+, 4.3) after incubation with a full spike peptide pool compared to healthy controls [%CD4^+^ CFSE-low, mean 1.3 (P<0.01); %CD8^+^, 0.4 (P<0.001)] (**Figure 4B**). Overall, more samples in the aCD20-MS group [CD4^+^,17/19 (89.5%); CD8^+^, 17/19 (89.5%)] were above the positivity threshold compared to the healthy control group [CD4^+^, 10/17 (58.8%); CD8^+^, 7/17 (41.2%)]. In conclusion, vaccinated aCD20-MS exposed to CoV-2 spike protein retain higher T-cell responses with increased cytokine release and potent expandability even in conditions of B-cell deficiency and compromised humoral response.

## 4 Discussion

Anti-CD20 monoclonal antibodies represent a form of B-cell-depleting therapy originally designed for the elimination of cancerous B-cells and now also extensively used to treat autoimmune diseases, including systemic lupus erythematosus, rheumatoid arthritis and multiple sclerosis. Although the mechanism of action is not completely known, anti-CD20 antibodies are thought to act by depleting memory B-cells and blocking a pathogenic function unrelated to antibody secretion (54). This is because plasma cells and plasmablasts are devoid of CD20 and spared from antibody-induced cytolysis and no substantial changes in antibody levels have been observed in blood of treated individuals (55, 56). In contrast, antibody titers generated against novel pathogens during anti-CD20 induced B-cell immunodepletion can be highly reduced compared to normal conditions. This acquired immunosuppression is thought to make antiCD20-treated individuals more susceptible to different forms of infection and less likely to develop immunoprotection upon vaccination.

As already observed by other groups, the present study finds decreased anti-spike (RBD) IgG antibody levels in aCD20-MS patients when compared to healthy controls. In samples collected during the first 6 months after vaccination with 2-dose mRNA-based vaccines, only 23.8% of aCD20-MS participants were able to generate antibody titers above the positive threshold. This seroconversion (SC) rate is similar to those observed in other published studies focused on the effect of anti-CD20 exposure in COVID-19 vaccinated patients with multiple sclerosis, hematopoietic malignancies, and rheumatological diseases (22, 57, 58). We also confirm that the lower antibody levels detected are insufficient to induce positive hACE2-RBD blocking activity. The aCD20-MS patients included here received the first vaccine inoculation at a median of 114.5 days [IQR: 81-138.5] after their last anti-CD20 infusion treatment. The elapsed time is shorter than reported in similar studies presenting higher SC rates (e.g (46), showing an SC rate > 60% and a median elapsed time of 174.3 days [IQR: 121.1-184.8]). This would suggest a prevalence of strong B-cell depletion in our test cohort. In fact, most of the patients had significantly reduced CD19^+^ B-cell levels (< 20 cells per µL) as measured close to sample collection. Interestingly, the individual in this study with highest detected IgG antibody titer and RBD-hACE2 blocking activity is the only one receiving COVID-19 immunization after the recommended 6-month period between anti-CD20 infusions (last infusion to 1^st^ dose vaccination interval time: 342 days). This patient, as well as other seroconverted aCD20-MS individuals, presented close to normal levels of CD19^+^ B-cells. These results suggest that specific vaccine regimens applied in close coordination with anti-CD20 treatment schedules and proper monitoring of B-cell repopulation might be needed for optimal seroconversion of aCD20-MS patients (59).

Even in the context of limited seroconversion, some aCD20-MS patients in this study were capable of developing anti-spike (RBD) IgG antibodies under conditions of strong B-cell aplasia. Severe B-cell depletion upon anti-CD20 therapy is only detected in peripheral blood and it is not known if germinal center reactions directed towards antibody production are still taking place in draining lymph nodes. Nevertheless, it would be of interest to study whether different operative pathways of antibody production are being used by these patients and analyze the quality of the humoral response as compared to individuals who seroconverted with normal B-cell count values. Interestingly, in addition to the depletion of peripheral B-cells in aCD20-MS, recent results also describe a significant reduction in circulating follicular helper CD4^+^ T-cell (TFH) responses (21). TFH cells are responsible for promoting the coordinated production of long-lasting and high-affinity antibodies by B-cells in germinal center reactions in response to infections or vaccinations (60, 61). The lower response in aCD20-MS patients would suggest an impact on the number and quality of antibodies being produced.

Contrary to the lower antibody titers, a higher polyfunctional spike-specific T-cell response in the aCD20-MS group compared to control was observed. These results support previous findings (21, 46, 47), but are also in contradiction to other published data showing no change or decrease (23, 48, 49). The inconsistency could potentially be explained by the different techniques used for the *ex vivo* detection of T-cell activation (including ELISpot and flow cytometry-based detection assays like Intracellular Cytokine Staining (ICS) and Activation-Induced Markers (AIM)). In addition to a higher cytokine release phenotype, an enhanced proliferative response after *ex vivo* spike stimulation of PBMCs derived from aCD20-MS patients was also detected. Moreover, a clear lack of correlation in the present study between both adaptive immune responses was obvious at the individual level (**Figure 3**). A similar decoupling effect has been observed in CoV-2 infected individuals with hematological cancer (especially those treated with anti-CD20 therapy), showing the generation of a T-cell response and disease control, even in the absence of neutralizing antibodies (44, 62). In a broader consideration, it is known that healthy convalescent COVID-19 patients with no seroconversion have a similar T-cell response compared to seropositive patients after the stimulation of PBMCs with CoV-2 spike and nucleocapsid peptide pools (63, 64).

Several potential reasons, contributing individually or in combination, may explain the higher T-cell responses observed in the aCD20-MS cohort. For example, it would be of interest to determine if a stronger response is linked to higher occurrence of a specific HLA configuration and/or higher presence of preexisting cross-reactive T-cells against endemic coronavirus (HCoV). The effect could also be due to the elimination of a subpopulation of B-cells with a T-cell inhibition function. More indirectly, possible alterations in the number of specific subtypes of T-cells due to population rearrangements under B-cell depleting conditions, maybe occurring in a MS immune-specific context, could also favor the higher T-cell response reported. Interestingly, significant changes in several T-cell populations have been observed during B-cell depletion and repopulation after therapy. For example, recent cellular profiles in antiCD20-treated patients have identified a higher presence of naïve T-cells (65, 66). It is conceivable that an increase in naïve T-cells could affect the amount of unique T-cell receptors available to detect new pathogens or antigens. Future studies should be focused on determining the immunological memory derived from the observed T-cell response by considering longitudinal time points, determining the effect of vaccine boosters or previous CoV-2 infections and identifying factors linked to higher chances for seroconversion or enhanced T-cell response in aCD20-MS patients.

Most importantly, it is hard to predict the real-world level of protection of aCD20-MS patients with an unbalanced adaptive immune system consisting of a defective humoral response but normal (or above normal) cellular response, as described here and by others. As mentioned in the introduction, a higher number of infections is likely to occur in vaccinated aCD20-MS patients as they lack the neutralizing antibodies necessary to block infection. A normal or enhanced T-cell response might suggest some level of protection against severe disease. Keeping track of the number and, more importantly, severity of CoV-2 breakthrough infection cases in the vaccinated MS community should give us a better idea of the level of vaccine effectiveness in patients under DMTs, including anti-CD20 treatment (67). In addition, the present study highlights the need to monitor vaccine-induced cellular response at the individual level, in addition to tracking antibody levels, for a better understanding of the benefits of current vaccination programs and the design of vaccinations in the future (68).

## 5 Limitations

There are a number of limitations associated with this study. First, the use of cryopreserved samples can be considered a technical limitation. Cryopreservation of PBMCs can affect cell viability, alter the detection of T-cell markers and the performance of T-cell responses (69–71). Nevertheless, the use of frozen cells allowed us to analyze multiple samples simultaneously making the comparisons presented in this study more reliable. Second, only individuals that received a two-dose COVID-19 vaccine regimen were included and, since immunocompromised patients were recommended for a third dose early during the United States vaccination campaign, the number of samples available was limited. Third, the healthy control group includes mostly BNT162b2-vaccinated individuals with almost no representation of mRNA-1273 recipients. Fourth, the study mainly considers samples from patients that were vaccinated less than six months after their last anti-CD20 infusion and with strong B-cell depletion. The inclusion of only one patient receiving vaccination past this time lapse limits our capacity to analyze the correlation between seroconversion and B-cell repopulation like the ones performed in other studies (21, 25, 72).

Finally, the significant age difference between groups is also of notice. Several publications considering COVID-19 mRNA-based vaccine responses in healthy individuals have observed a slight decrease in antibody production and no change in T-cell responses with age (6, 73, 74). Another study showed lower antibody levels and neutralizing potency, as well as decreased production of IFNγ and IL-2 by spike-specific T-cells in individuals over 80 years of age (75). It is possible that some level of response impairment due to an aging immune system occurs in some of the oldest participants included in the present study. Nevertheless, no correlation between age and anti-RBD IgG antibody titers or T-cell response was observed for the aCD20-MS group.

## Data availability statement

The original contributions presented in the study are included in the article/**Supplementary Material**. Further inquiries can be directed to the corresponding author.

## Ethics statement

The studies involving human participants were reviewed and approved by Western Institutional Review Board (WIRB). The patients/participants provided their written informed consent to participate in this study.

## Author contributions

Conceptualization, RA-D, JeL and SAS. Methodology, RA-D, JeL, VK, JoL, GF, MM, JiL, MR, and SAS. Investigation, RA-D, JeL and SAS. Writing – Original Draft, RA-D. Writing – Review and Editing, RA-D, JeL, VK, JLei, GF, MM, JiL, MR, and SAS. Funding Acquisition, SAS. Supervision, RA-D, JeL and SAS. All authors contributed to the article and have seen and approved the submitted version.

## Funding

This study was funded by internal Institutional grants.

## Acknowledgments

We would like to thank the study participants for their generosity and for making this study possible. We thank nurses at IMSMP Infusion Suite for their help collecting samples.

## Conflict of interest

The authors declare that the research was conducted in the absence of any commercial or financial relationships that could be construed as a potential conflict of interest.

## Publisher’s note

All claims expressed in this article are solely those of the authors and do not necessarily represent those of their affiliated organizations, or those of the publisher, the editors and the reviewers. Any product that may be evaluated in this article, or claim that may be made by its manufacturer, is not guaranteed or endorsed by the publisher.
