# Supplementary figures and images for: Strong T-cell activation in response to COVID-19 vaccination in multiple sclerosis patients receiving B-cell depleting therapies
Source: Front Immunol. 2022 Aug 5;13:926318. doi: 10.3389/fimmu.2022.926318 (PMC9388928; doi:10.3389/fimmu.2022.926318)

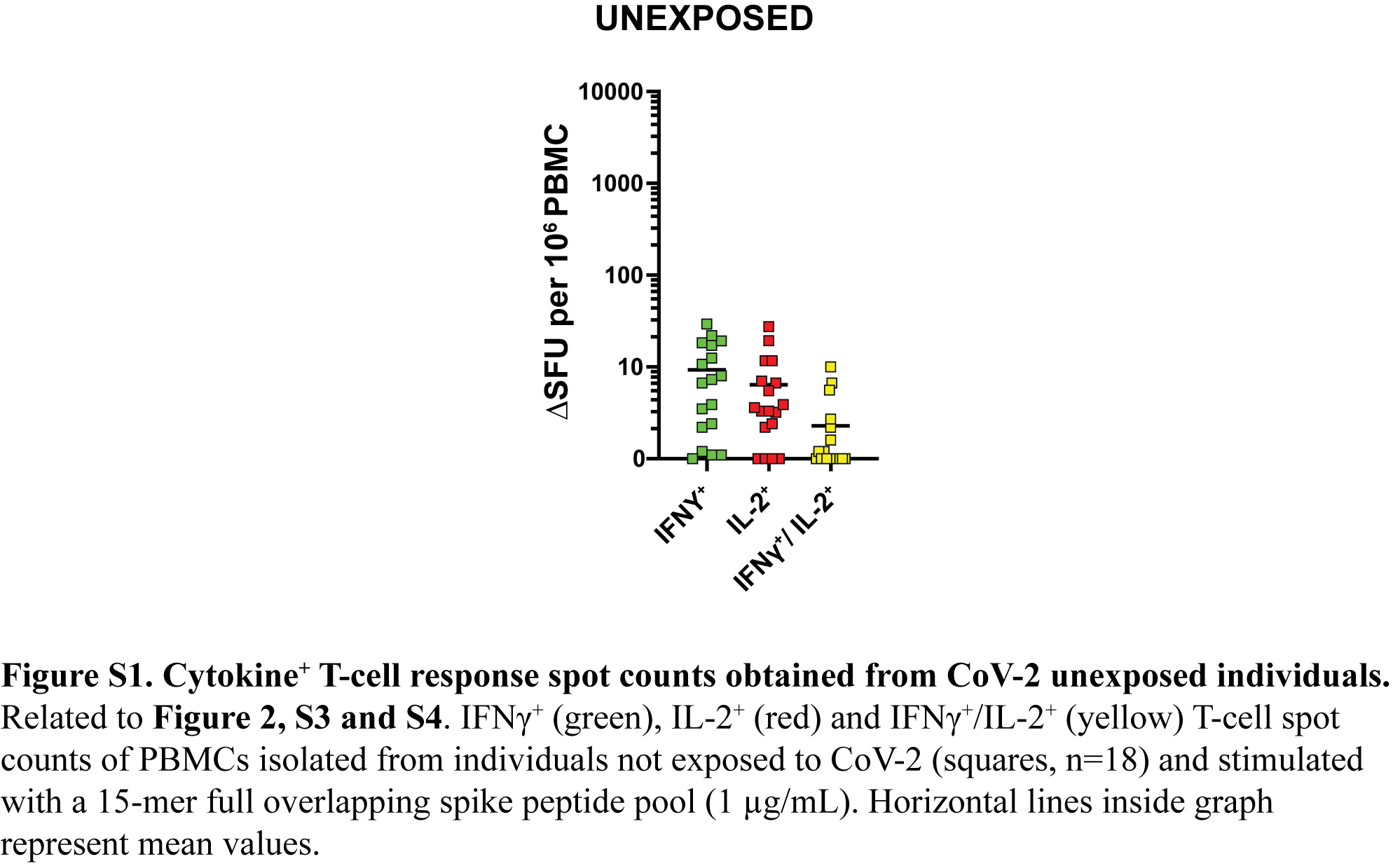

Supplement: Supplementary file 1 [file Image_1.jpeg]

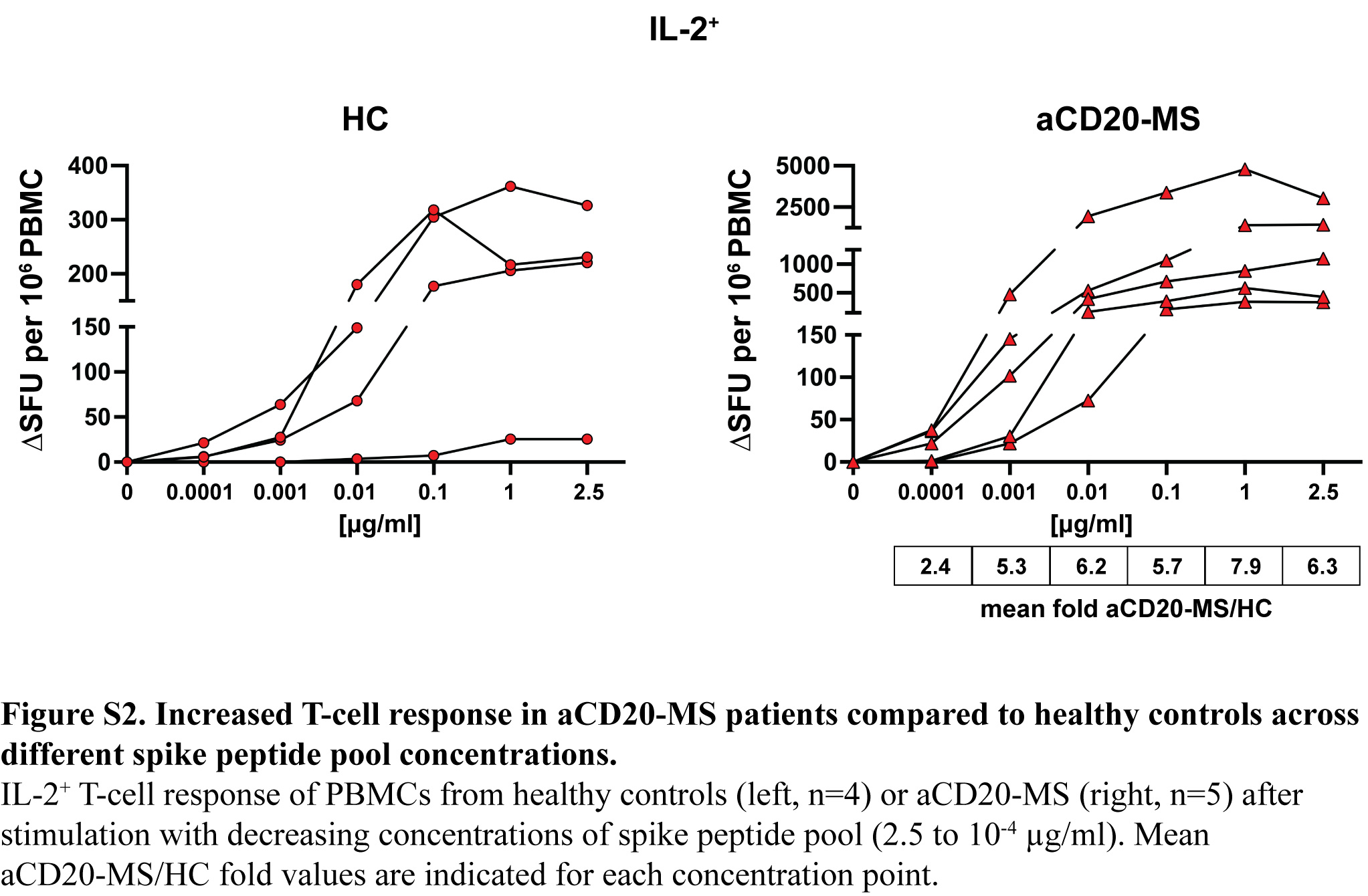

Supplement: Supplementary file 2 [file Image_2.jpeg]

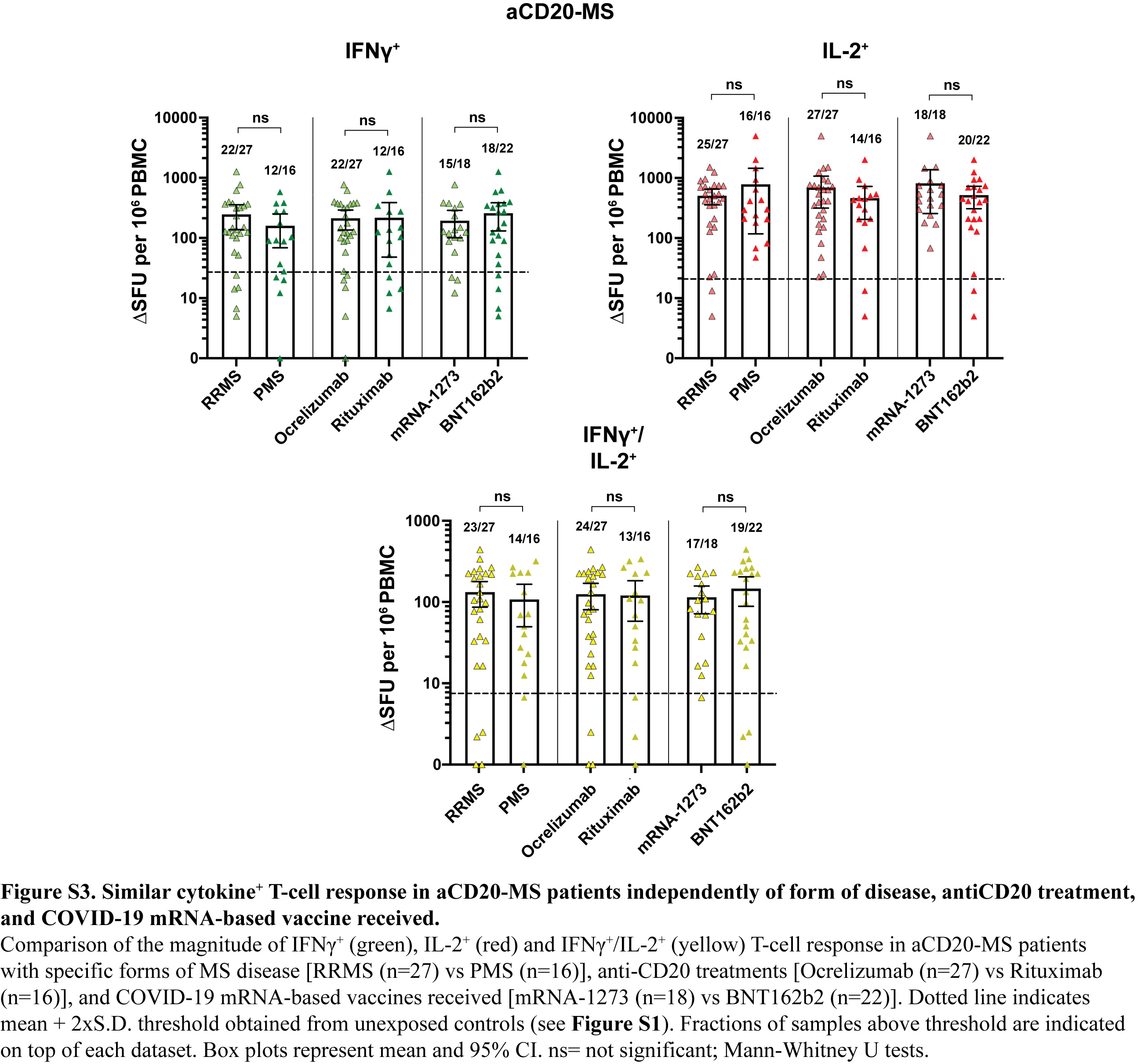

Supplement: Supplementary file 3 [file Image_3.jpg]

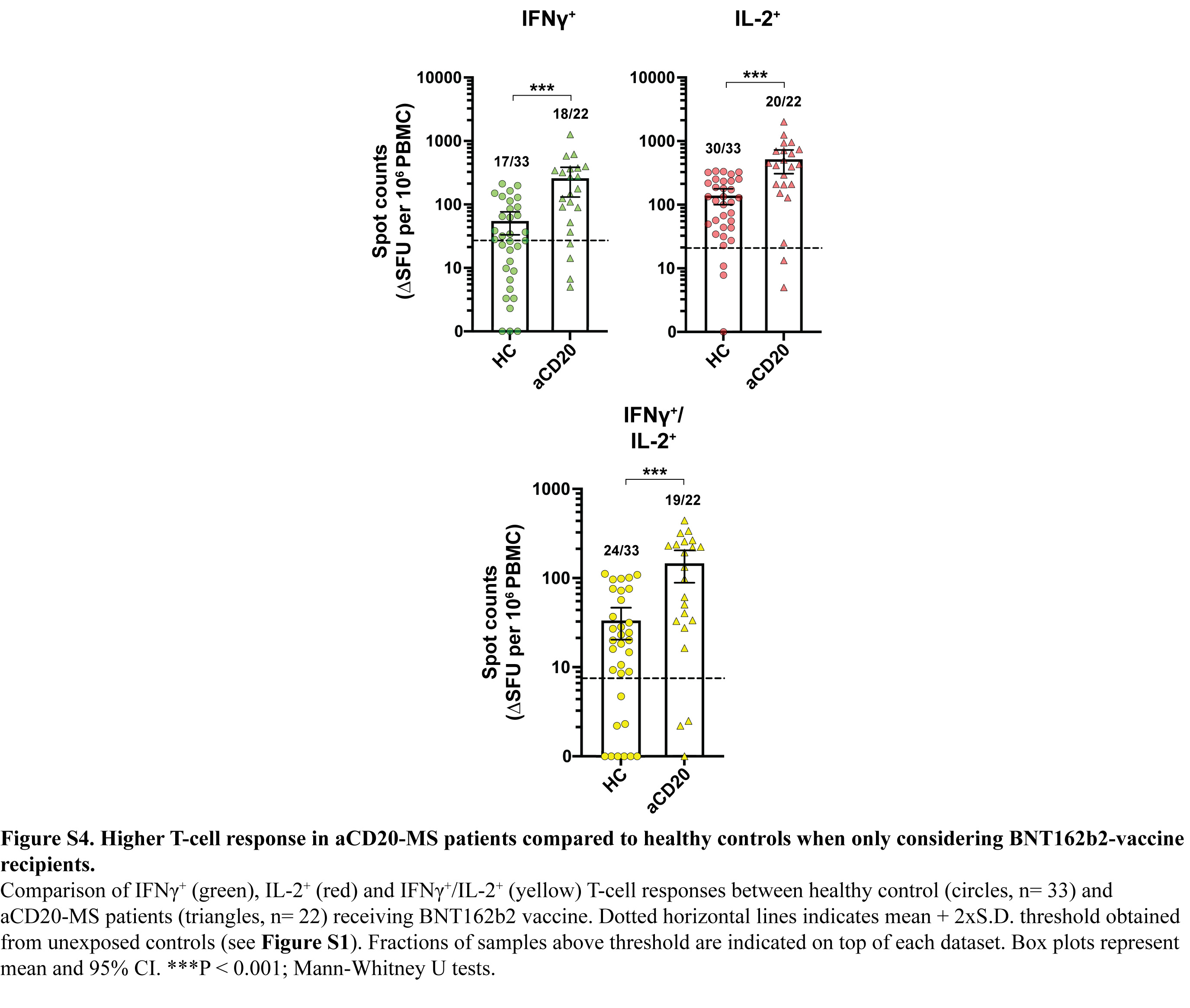

Supplement: Supplementary file 4 [file Image_4.jpeg]

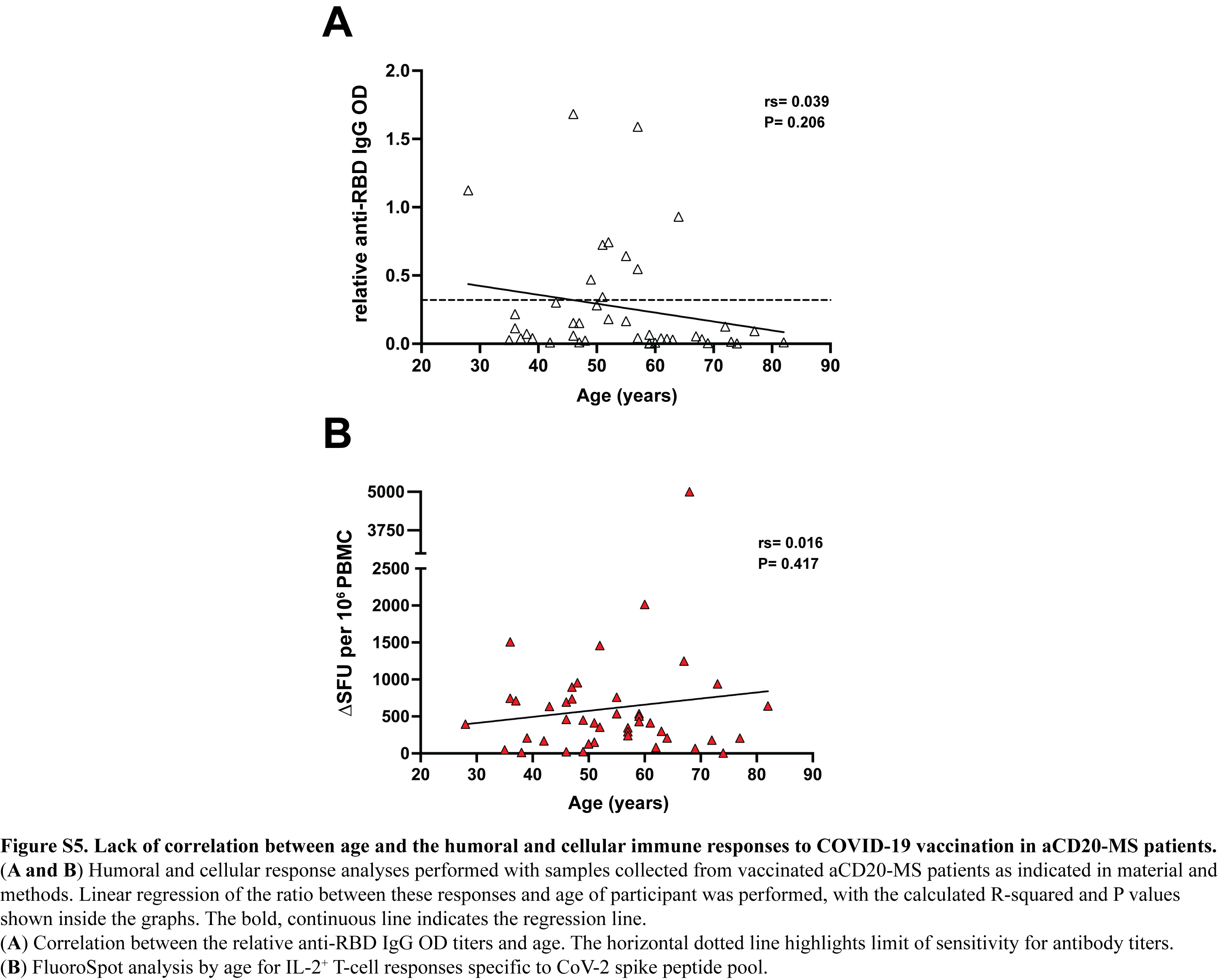

Supplement: Supplementary file 5 [file Image_5.jpg]

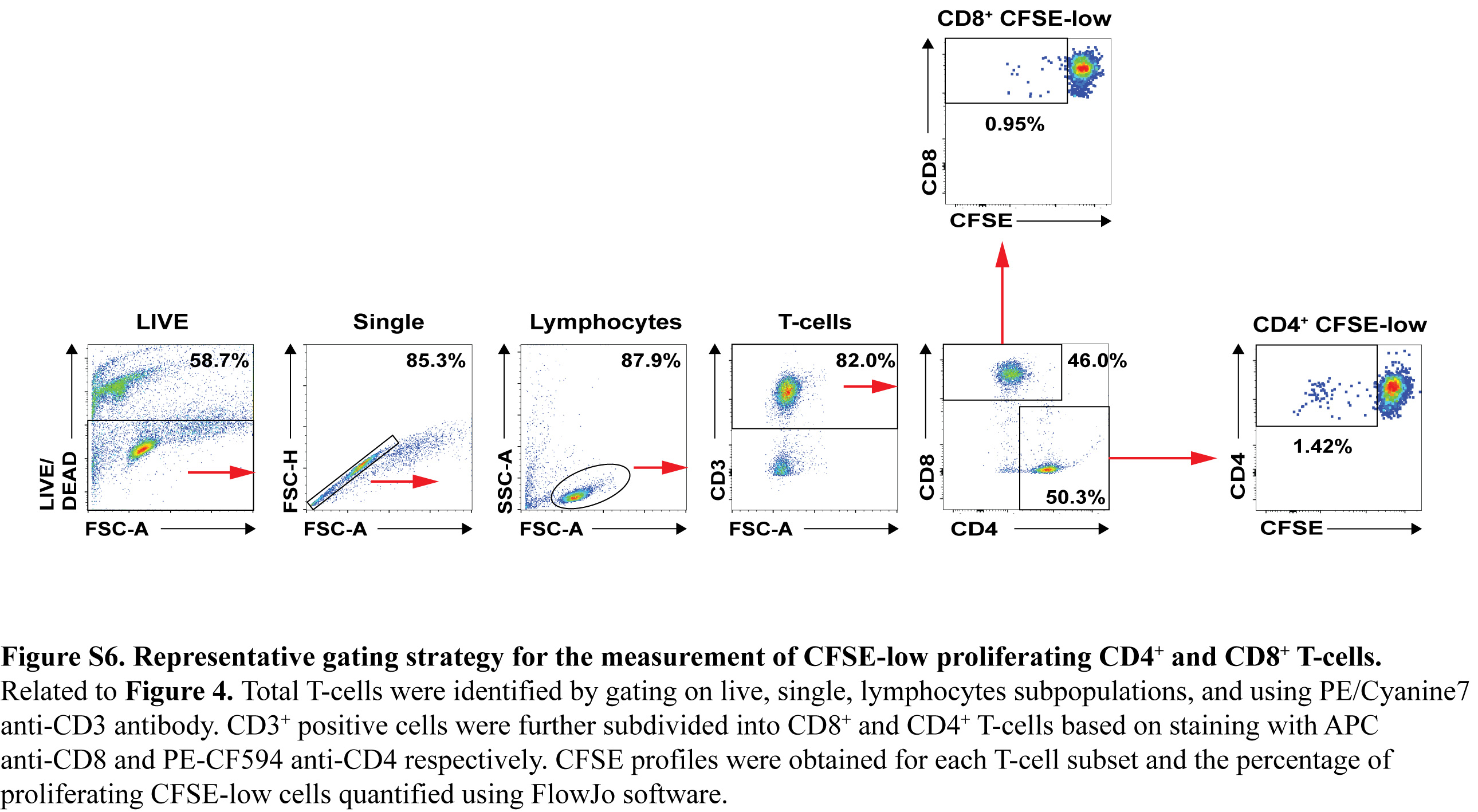

Supplement: Supplementary file 6 [file Image_6.jpeg]
